# Three cases of macular hole that occurred in inferior scleral staphyloma associated with tilted disc syndrome: a case series

**DOI:** 10.1186/s13256-022-03252-7

**Published:** 2022-01-29

**Authors:** Hiroshi Mizuno, Hiroyuki Suzuki, Masashi Mimura, Masanori Fukumoto, Takaki Sato, Teruyo Kida, Tsunehiko Ikeda

**Affiliations:** 1 Department of Ophthalmology, Osaka Medical and Pharmaceutical University, Takatsuki, Osaka Japan; 2 Department of Ophthalmology, Hirakata City Hospital, Hirakata, Osaka Japan; 3 Department of Ophthalmology, First Towakai Hospital, Takatsuki, Osaka Japan; 4 Fukumoto Eye Clinic, Kadoma, Osaka Japan; 5 Department of Ophthalmology, Osaka Kaisei Hospital, 1-6-10 Miyahara Yodogawa-ku, Osaka, 532-0003 Japan

**Keywords:** Macular hole, Inferior posterior staphyloma, Tilted disc syndrome, Pars plana vitrectomy, Optical coherence tomography

## Abstract

**Background:**

The objective is to examine the clinical characteristics of three patients with macular hole that occurred in inferior posterior staphyloma associated with tilted disc syndrome.

**Case presentations:**

This study involved three eyes of three Japanese female patients (mean age 76.0 years, range 73–84 years) with macular hole occurring in inferior posterior staphyloma associated with tilted disc syndrome. One of the three eyes was slightly myopic, while the other two eyes were highly myopic. In all three eyes, the macular hole was found to be located in or near the margin of the inferior posterior staphyloma. In one eye, the extent of retinoschisis was rather broad in the margin of the macular hole, and another eye had a history of treatment for choroidal neovascularization. As surgical treatment, the internal limiting membrane in areas surrounding the macular hole was detached after producing artificial posterior vitreous detachment, and a gas tamponade was performed. It was found during surgery that the extensibility of the retina in the margin of the MH was decreased in the three eyes as compared with a usual macular hole. Although the macular hole was successfully closed in all three cases post surgery, the layer structure of the central retina was poorly repaired in all three cases and choroidal neovascularization remained in one case. In all three cases, corrected visual acuity remained at 0.3–0.5 post surgery.

**Conclusions:**

Our findings showed poor improvement of visual acuity in all three cases post surgery, even if closure of the macular hole is achieved, thus suggesting that in cases of macular hole associated with tilted disc syndrome and inferior posterior staphyloma, the presence of macular dysfunction existing prior to the onset of macular hole affects visual prognosis.

## Introduction

Tilted disc syndrome (TDS) is a congenital anomaly of the optic nerve that develops due to a failed closure of the optic fissure in the embryonic stage, and its frequency is reportedly high among cases with congenital anomalies of the optic nerve [1, 2]. In the quadrant of the TDS margin where the conus is present, there is localized posterior staphyloma, usually on the inferior or inferotemporal side. In cases where the margin of interior posterior staphyloma (IPS) splits the macular region, macular complications such as serous retinal detachment (SRD) [3–9], choroidal neovascularization (CNV) [10–13], retinoschisis (RS) [14], and retinal pigment epithelium atrophy [15] often occur. IPS is a feature that is common to both high myopia and TDS. However, the concomitant occurrence of a macular hole (MH) has rarely been reported [16–18]. Here, we examined the clinical characteristics and postoperative outcomes of vitreous surgery in three patients with MH that occurred in IPS associated with TDS.

## Case presentations

### Patient 1

**Fig. 1:**
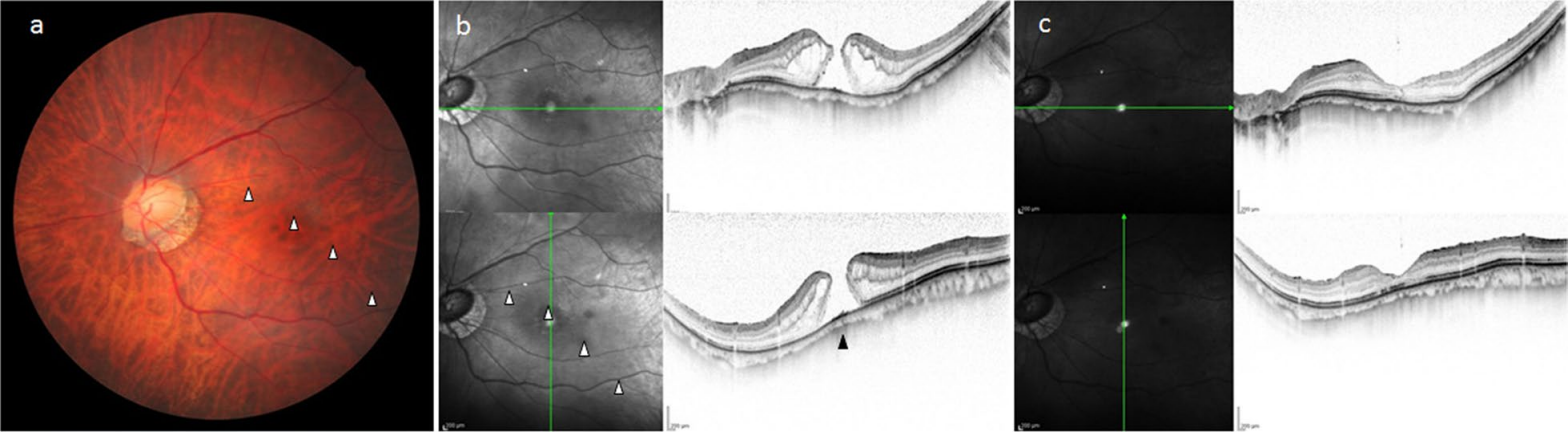
**a** Fundus image of the left eye in case 1 obtained prior to vitreous surgery. Tilted disc syndrome (TDS) accompanied by conus on the inferior temporal side of the optic disc and shallow interior posterior staphyloma (IPS) on the inferior to inferior-temporal side can be seen. The boundary sites in regard to the IPS limit in the fundus image are indicated by white arrowheads. **b** Optical coherence tomography (OCT) image of the left eye in case 1 obtained prior to vitreous surgery. Full-thickness macular hole (MH) with retinoschisis (RS) can be seen, with the RS spread rather more extensively on the IPS side. The boundary sites in regard to the IPS limit in the OCT image are indicated by white arrowheads. The choroidal thinning at the boundary site in regard to the IPS limit in the OCT image is indicated by a black arrowhead. **c** OCT image of the left eye in case 1 obtained post vitreous surgery. Post surgery, the MH was closed; however, the ellipsoid zone was slightly irregular and the layer structure of central retina was poorly repaired at 1 year postoperatively

Patient 1 was a 73-year-old Japanese female who visited our department after becoming aware of reduced visual acuity (VA) in her left eye. Both of her eyes were pseudophakic, and the VA in her right and left eye was right vision (RV) = (1.2 × S − 2.50D C − 0.50D Ax90°) and left vision (LV) = (0.4 × S − 2.50D C − 3.00D Ax80°), respectively. In both eyes, she had myopia of −5 diopters (D) before cataract surgery, and the corrected VA of the left eye before the onset of MH was 1.0D. In the fundus of the left eye, there was TDS accompanied by conus on the inferior temporal side of the optic disc, with shallow IPS present on the inferior to inferior-temporal side (Fig. 1a). Optical coherence tomography (OCT) revealed a full-thickness MH, with RS occurring in the margin of the MH. The RS spread rather more extensively on the IPS side (Fig. 1b). Choroidal thinning in regard to the boundary site of the IPS limit was observed in the OCT image. Thus, pars plana vitrectomy (PPV) was performed for treatment of the MH. It was found during surgery that the posterior vitreous body was not detached, and artificial posterior vitreous detachment was produced with a diamond scraper from the posterior pole to marginal areas after applying triamcinolone. Brilliant Blue G was then applied to areas around the MH to stain the internal limiting membrane (ILM), and the ILM around the MH was detached with vitreous forceps. The margin of the MH was then pulled centripetally with a backflush needle, which revealed that the extensibility of the retina in the MH margin was slightly decreased compared with usual cases. Subsequently, simultaneous replacement of intraocular fluid and air and gas tamponade with 20% sulfur hexafluoride (SF_6_) were performed, with the patient then being instructed to remain in a prone position. Although the MH was found to be successfully closed post surgery, an OCT examination revealed that the ellipsoid zone was slightly irregular and that the layer structure of the central retina was poorly repaired (Fig. 1c). At 1 year postoperatively, the corrected VA in her left eye remained at 0.5D.

### Patient 2

**Fig. 2:**
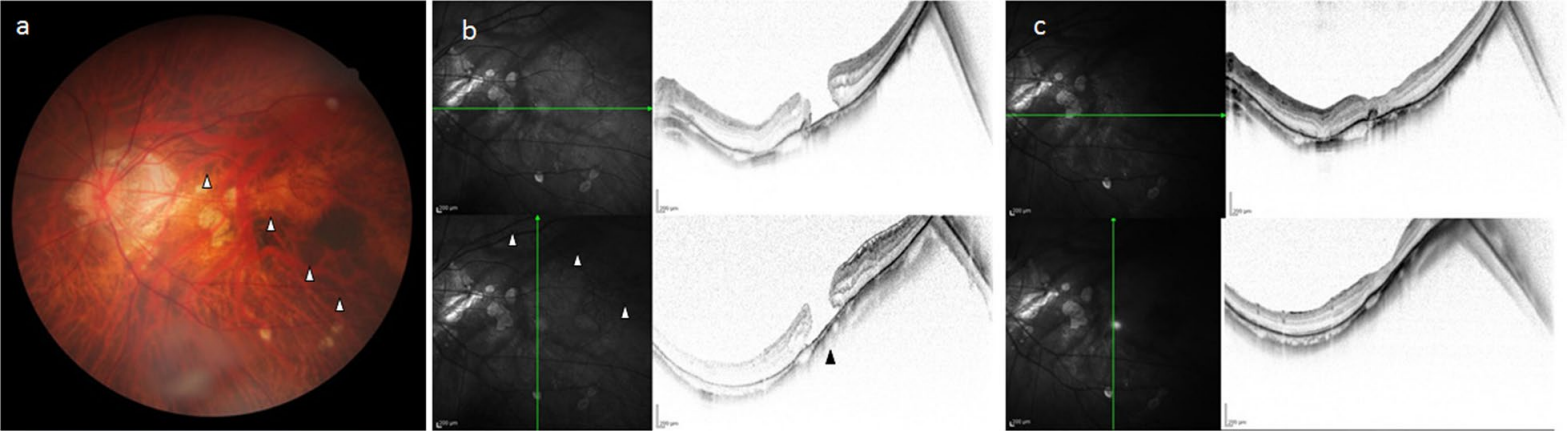
**a** Fundus image of the left eye in case 2 obtained prior to vitreous surgery. TDS and IPS can be seen on the inferior to inferior-temporal side of the ocular fundus. The boundary sites in regard to the IPS limit in the fundus image are indicated by white arrowheads. **b** OCT image of the left eye in case 2 obtained prior to vitreous surgery. Full-thickness MH with myopic choroidal neovascularization (m-CNV) and epiretinal membrane can be seen. The boundary sites in regard to the IPS limit in the OCT image are indicated by white arrowheads. The choroidal thinning in regard to the boundary site of the IPS limit in the OCT image is indicated by a black arrowhead. **c** OCT image of the left eye in case 2 obtained post vitreous surgery. Central retinal thickness was slightly thin and m-CNV remained at 1 year postoperatively

Patient 2 was an 80-year-old Japanese female who was found to have myopic choroidal neovascularization (m-CNV) in her left eye at a neighborhood clinic, and was subsequently referred to our department. Both of her eyes were pseudophakic, and the VA in her right and left eye was RV = (0.6 × S − 0.50D C − 0.75D Ax110°) and LV = (0.3 × S + 0.75D C − 0.75D Ax180°), respectively. The refraction before cataract surgery was −14D in the right eye and −12D in the left eye, showing high myopia. The VA in her left eye was 0.7D before the onset of m-CNV. Intravitreal injection of aflibercept resulted in a reduction of m-CNV, but thereafter MH occurred, resulting in a decrease in VA to 0.2D. TDS and IPS were found on the inferior to inferior-temporal side of the ocular fundus (Fig. 2a), and OCT revealed a full-thickness MH with m-CNV and epiretinal membrane (Fig. 2b). Choroidal thinning in regard to the boundary site of the IPS limit was observed in the OCT image. Thus, PPV was subsequently performed for treatment of the MH. It was found during surgery that the posterior vitreous body was not detached, and the extensibility of the retina in the MH margin was slightly decreased, the same as in case 1. Posterior vitreous detachment, ILM detachment, and gas tamponade were performed. Although the MH was found to be successfully closed post surgery, an OCT examination at 1 year postoperatively revealed that the central retinal thickness was slightly thin and that m-CNV remained (Fig. 2c). One year postoperatively, the corrected VA in her left eye remained at 0.5D.

### Patient 3

**Fig. 3:**
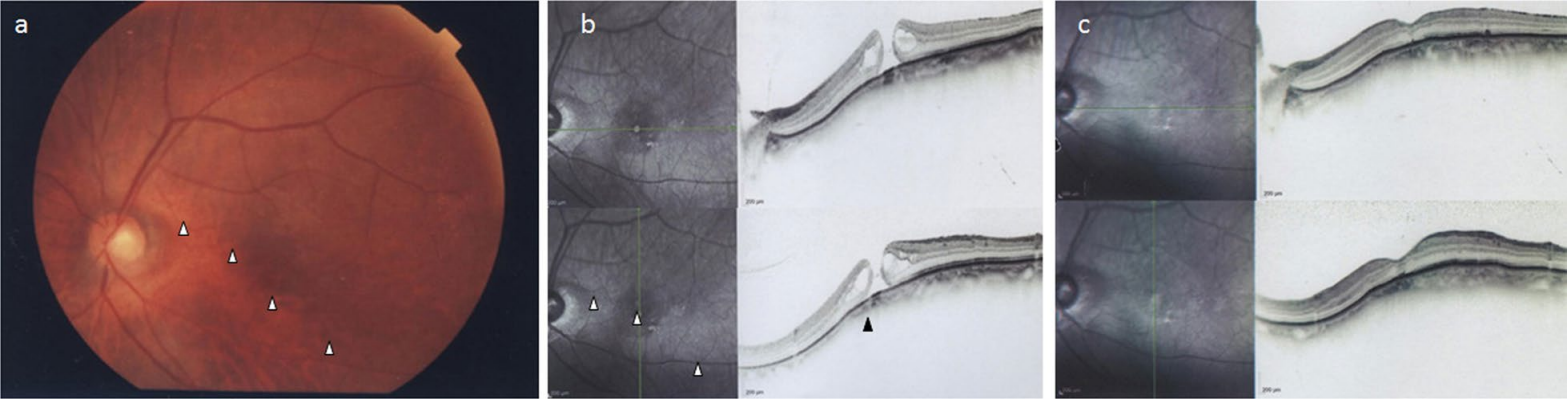
**a** Fundus image of the left eye in case 3 obtained prior to vitreous surgery. TDS and IPS can be seen on the inferior to inferior-temporal side of the ocular fundus. The boundary sites in regard to the IPS limit in the fundus image are indicated by white arrowheads. **b** OCT image of the left eye in case 3 obtained prior to vitreous surgery. Full-thickness MH can be seen. The boundary sites in regard to the IPS limit in the OCT image are indicated by white arrowheads. The choroidal thinning in regard to the boundary site of the IPS limit in the OCT image is indicated by a black arrowhead. **c** OCT image of the left eye in case 3 obtained post vitreous surgery. Minor derangement of the layer structure of the central fovea remained at 1 year postoperatively

Patient 3 was a 75-year-old Japanese female who visited our department with the primary complaint of decreased VA in her left eye. Both of her eyes were pseudophakic, and the VA in her right and left eye was RV = (0.4 × S−0.70D C − 1.75D Ax100°) and LV = (0.2 × S − 1.50D), respectively. Refraction before cataract surgery was −8D, indicating high myopia, and the corrected VA in her left eye was 0.7D before the onset of MH. TDS and IPS were found on the inferior to inferior-temporal side of the ocular fundus in the left eye (Fig. 3a), and OCT revealed a full-thickness MH (Fig. 3b). Choroidal thinning in regard to the boundary site of the IPS limit was observed in the OCT image. In this case, the MH appeared to be inside a large IPS and not at the edge of the IPS, however, with also choroidal thinning at the limit of the MH. Thus, PPV was performed. Intraoperative findings were similar to those in case 1 and case 2. The MH margin was slightly pulled toward the IPS, thus forming the MH into an elliptic shape. Although the MH was found to be successfully closed post surgery, an OCT examination at 1 year postoperatively revealed a remaining minor derangement of the layer structure of the central fovea (Fig. 3c). At 1 year postoperatively, the corrected VA in her left eye remained at 0.3D.

## Discussion

TDS is a frequent congenital anomaly of the optic nerve that develops due to failed closure of the optic fissure in the embryonic stage [1]. In TDS cases, the optic disc is inclined downward, and there is often a conus inferior or inferotemporal to the optic disc, with IPS present in that direction [2]. It is known that IPS is a feature that is common to both high myopia and TDS, and in cases where the margin of the IPS splits the macular region, SRD, CNV, and RS are likely to occur, due to the fact that the Bruch's membrane in the boundary area is fragile [3–15]. Ellabban *et al*. [19] reported that swept-source OCT of the macular region in IPS revealed marked thinning of the choroid membrane in an area where the IPS margin split the macular region, making the occurrence of SRD and CNV more likely under the influence of age-related changes. Maruko *et al*. [20] also reported that thinning of the choroid membrane would cause occlusion of the choriocapillary layer, and disturbance of choroid circulation would induce SRD and CNV.

Although it is generally well known that high myopia is often complicated with an MH, it has rarely been reported that IPS associated with TDS is complicated with an MH. In fact, to the best of our knowledge, there have been only three published reports of such cases. Cohen *et al*. [16]. reported observing some sort of macular complication in 71 (77.1%) of 92 eyes in patients with TDS. In that study, they reported that frequent complications included pigment epithelial disorder occurring in 34 eyes (36.9%), CNV in 24 eyes (26%), and SRD in 16 eyes (17.3%), while split-thickness MH was present in 3 eyes (3.2%). In a report by Coco *et al*. [17]_,_ the authors examined 68 eyes for which OCT revealed dome-shaped macula or IPS, and found MH in 2 eyes with dome-shaped macula and 1 eye with IPS. However, the authors made no reference to the PPV results in that study. Bruè *et al*. [18] reported a case of TDS-related MH that closed spontaneously during the course of illness, yet no mention was made in that study about the relationship between TDS and MH.

It has previously been reported that the rate of postoperative closure of MH is lower in cases of high myopia than in cases of non-high myopia [21]. This may be explained by tangential traction of the retina due to posterior staphyloma in addition to the thinning of the retina. In cases of IPS, thinning and tangential traction of the retina do not occur in the entire posterior fundus, and thus, the occurrence of MH may be rare even in cases of high myopia. However, in case 3 in this study, the MH margin was pulled toward the IPS, thus forming the MH into an elliptic shape. Hence, it is possible that the shape of the IPS is involved in the occurrence of MH.

Although the MH was successfully closed post surgery in the three cases presented here, the layer structure of central retina was poorly repaired at 1 year postoperatively in all cases. In addition, the VA at 1 year postoperatively remained between 0.3D and 0.5D, thus illustrating that the VA did not completely recover to the level prior to the onset of MH. It is possible that, in cases of MH associated with TDS and IPS, traction from IPS acts on the central retina, causing thinning of the retina, the same as in eyes with posterior staphyloma associated with usual high myopia. In addition, since SRD often occurs in cases of MH associated with TDS and IPS, gradual development of SRD may be present before the onset of MH, thus inducing gradual deterioration of central retinal function. In case 1, the preoperative OCT examination showed slightly more extensive RS on the IPS side in comparison with typical MH cases, thus suggesting that RS might have already been present in the central fovea prior to the onset of MH. In case 2, there was concomitant CNV before the onset of MH. Although anti-vascular endothelial growth factor therapy resulted in the reduction of CNV, an OCT examination performed after PPV revealed a small amount of CNV remaining, which might have affected the visual prognosis.

## Conclusions

Unfortunately, accurate time-course changes in the pathological status of the present three cases before the onset of MH were not obtained. Regardless, it is possible that macular complications such as SRD, CNV, and RS may be present before the onset of an MH when the MH occurs in association with TDS and IPS. Therefore, it is vital to keep in mind the possibility that improvement in vision can be poor even if the MH is closed post surgery.

## Data Availability

The datasets used in the current study are available from the corresponding author on reasonable request.

## Abbreviations

TDS: Tilted disc syndrome
IPS: Interior posterior staphyloma
SRD: Serous retinal detachment
CNV: Choroidal neovascularization
RS: Retinoschisis
MH: Macular hole
VA: Visual acuity
D: Diopters
OCT: Optical coherence tomography
PPV: Pars plana vitrectomy
ILM: Internal limiting membrane
m-CNV: Myopic choroidal neovascularization
